# Nutrition and adult neurogenesis in the hippocampus: Does what you eat help you remember?

**DOI:** 10.3389/fnins.2023.1147269

**Published:** 2023-02-23

**Authors:** Sonia Melgar-Locatelli, Marialuisa de Ceglia, M. Carmen Mañas-Padilla, Celia Rodriguez-Pérez, Estela Castilla-Ortega, Adriana Castro-Zavala, Patricia Rivera

**Affiliations:** ^1^Instituto de Investigación Biomédica de Málaga y Plataforma en Nanomedicina-IBIMA Plataforma BIONAND, Málaga, Spain; ^2^Departamento de Psicobiología y Metodología de las Ciencias del Comportamiento, Facultad de Psicología, Universidad de Málaga, Málaga, Spain; ^3^UGC Salud Mental, Hospital Universitario Regional de Málaga, Málaga, Spain; ^4^Departamento de Nutrición y Bromatología, Facultad de Ciencias de la Salud, Universidad de Granada, Granada, Spain; ^5^Instituto de Nutrición y Tecnología de los Alimentos ‘José Mataix’, Universidad de Granada, Granada, Spain; ^6^Instituto de Investigación Biosanitaria ibs.GRANADA, Granada, Spain

**Keywords:** adult neurogenesis, hippocampus, nutrients, diet, perinatal programming, gut microbiota

## Abstract

Neurogenesis is a complex process by which neural progenitor cells (NPCs)/neural stem cells (NSCs) proliferate and differentiate into new neurons and other brain cells. In adulthood, the hippocampus is one of the areas with more neurogenesis activity, which is involved in the modulation of both emotional and cognitive hippocampal functions. This complex process is affected by many intrinsic and extrinsic factors, including nutrition. In this regard, preclinical studies performed in rats and mice demonstrate that high fats and/or sugars diets have a negative effect on adult hippocampal neurogenesis (AHN). In contrast, diets enriched with bioactive compounds, such as polyunsaturated fatty acids and polyphenols, as well as intermittent fasting or caloric restriction, can induce AHN. Interestingly, there is also growing evidence demonstrating that offspring AHN can be affected by maternal nutrition in the perinatal period. Therefore, nutritional interventions from early stages and throughout life are a promising perspective to alleviate neurodegenerative diseases by stimulating neurogenesis. The underlying mechanisms by which nutrients and dietary factors affect AHN are still being studied. Interestingly, recent evidence suggests that additional peripheral mediators may be involved. In this sense, the microbiota-gut-brain axis mediates bidirectional communication between the gut and the brain and could act as a link between nutritional factors and AHN. The aim of this mini-review is to summarize, the most recent findings related to the influence of nutrition and diet in the modulation of AHN. The importance of maternal nutrition in the AHN of the offspring and the role of the microbiota-gut-brain axis in the nutrition-neurogenesis relationship have also been included.

## 1. Introduction

Hippocampus, a region extensively known to regulate learning, memory, and mood can be found among the highly sensitive, environment-responsive structures of the brain (Squire, 1992; Clelland et al., 2009; Castilla-Ortega et al., 2011; Murphy et al., 2014). The hippocampus is one of the main structures in the adult brain where the formation of new-born neurons, or neurogenesis, persists all lifelong (Eriksson et al., 1998). In this regard, the birth and maturation of neurons in the dentate gyrus of the adult hippocampus, known as adult hippocampal neurogenesis (AHN), is one of the most studied neuroplastic phenomena. Research in rodents supports a relevant role of AHN in the adaptation to environmental demands. For example, AHN facilitates a variety of cognitive functions including the long-term consolidation of new hippocampal memories -as well as the forgetting of previously acquired ones- and it is also involved in emotional regulation such as in providing stress resilience [reviewed in Wu et al. (2021)]. Accordingly, manipulations of AHN have functional consequences as they usually correlate with changes in memory and mood (Castilla-Ortega et al., 2011; Yau et al., 2015).

The plasticity of these new hippocampal neurons is greater than those of adult neurons. They exhibit a lower threshold for induction of long-term potentiation (LTP), possibly because new neurons can depolarize using currents of very small amplitude (Wang et al., 2000; Schmidt-Hieber et al., 2004). In addition, they are able to more rapidly form branches and synapses (Gould and Gross, 2002), so the plasticity of these new neurons is greater than those of more adult neurons. From 3-4 weeks of age the new neurons show similar properties to the older granular neurons that compose the cell layer of the dentate gyrus. This plasticity and excitability exhibited by new hippocampal neurons seem to contribute to explain the important role of the NHA in hippocampal-dependent learning and memory, such as spatial learning or complex associative learning (Leuner et al., 2006; Kee et al., 2007; Koehl and Abrous, 2011). Therefore, the processes of proliferation, survival, maturation, and functional integration of the new hippocampal neurons critically depend on intrinsic and extrinsic factors. Thus, aging, neuroinflammation, oxidative stress and brain injury, as well as exposure to drugs of abuse such as alcohol and opiates, negatively affect adult neurogenesis [as reviewed in Poulose et al. (2017)]. On the contrary, voluntary running or enriched environment are associated with enhanced AHN and with improvement of learning and memory (Fabel et al., 2009; Zainuddin and Thuret, 2012; Poulose et al., 2017; Kempermann, 2019). At the molecular level, modulation of AHN is mediated trough several signaling factors, including neurotrophic factors, transcriptional programs, inflammatory cytokines, neurotransmitters and hormones (Shohayeb et al., 2018), that are triggered by environmental demands. Many neurotrophic factors stimulate the activation of tropomyosin-related kinase (Trk) receptors, in turn activating intracellular signaling cascades that regulate NSC proliferation and fate. The role of neurotrophins and, in particular, of brain-derived neurotrophic factor (BDNF) in adult neurogenesis has been the subject of numerous studies; thus, BDNF-TrkB signaling positively regulates AHN and its dysregulation is associated with psychiatric and neurodegenerative disorders (Reichardt, 2006; Shohayeb et al., 2018).

Along with all these factors, over the last years emerged studies linking nutrition to AHN and mental health. Current literature suggests that dietary modifications can influence learning and memory as well as cognition and mood; so nutritional changes may be an inexpensive and relevant adjuvant intervention to boost mental and brain function (Zainuddin and Thuret, 2012; Murphy et al., 2014). Since neurogenesis can potentially regulate brain cognition and neuronal plasticity, nutritional factors that enhance neurogenesis may be attractive therapeutic targets for improving cognitive function and regulating different neurodegenerative and neuropsychiatric disorders [reviewed in Lim et al. (2021)].

**FIGURE 1:**
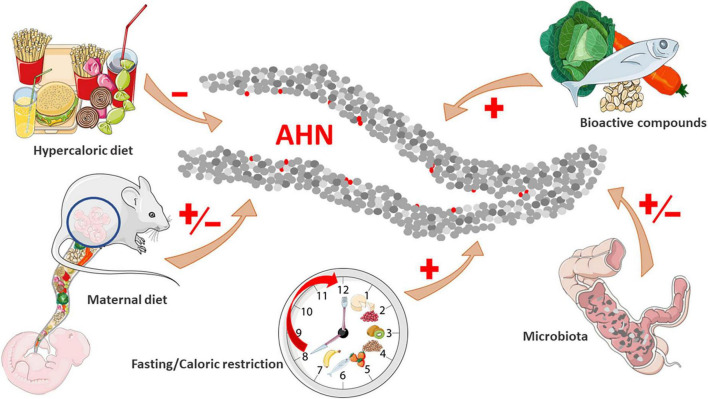
Impact of nutrition on adult hippocampal neurogenesis.

In this mini-review, we summarize the existing scientific literature on the influence of nutrition and diet on AHN (Figure 1), as a possible mechanism by which nutrition impacts on cognition and mental health. It is important to consider that, while studies in humans have demonstrated that certain nutrients and diets modulate cognition and behavior, researching AHN in human samples is elusive due to current methodological limitations (Moreno-Jiménez et al., 2021). Therefore, the evidence linking nutrition and AHN that composes this review comes mainly from preclinical studies performed in rats and mice.

## 2. Nutrition as a regulatory factor in AHN

### 2.1. High-calorie diets and bioactive compounds

Learning and memory, closely related to AHN, can be influenced by diet during development and into adulthood; there is an inverse correlation between the quality of the diet and the disorders associated with these hippocampal abilities (Zainuddin and Thuret, 2012; Murphy et al., 2014). In this context, high-calorie diets (HCD), characterized in rodents by high levels of saturated fats (40–60% fat) and/or refined sugars for at least 4 weeks, strongly impairs in a sex specific manner AHN decreasing proliferating cells, differentiated neuroblasts/immature neurons and mature neurons; and has been associated with disfunctions in hippocampal-dependent memory and neuroinflammation, (Park et al., 2010; van der Borght et al., 2011; Hsu et al., 2015; Pérez-Garciá et al., 2016; Robison et al., 2020; Kim et al., 2021; Paulo et al., 2021; Fierros-Campuzano et al., 2022). Most of these studies affirm that the downregulation of the neurotrophin BDNF and its signaling through cAMP response element-binding (CREB) and TrkB as the main mechanism involved in HCD-induced AHN injury (Molteni et al., 2002; Hwang et al., 2008; White et al., 2009; Kim et al., 2021; Paulo et al., 2021). Other authors highlight the increase of serum corticosterone, malondialdehyde (MDA) and tumor necrosis factor alpha (TNFα) as responsible for this decrease in AHN induced by HFD (Lindqvist et al., 2006; Park et al., 2010; van der Borght et al., 2011).

Diets enriched with bioactive compounds, such as omega-3 fatty acids, vitamins and polyphenols (presents on blueberries, olive oil, coffee, cocoa, tea and curcumin, among others) enhance AHN (Sarubbo et al., 2018; Rajaram et al., 2019; Dias et al., 2021). Omega-3 fatty acids (ω-3 FAs) -present in fish, vegetable oils, nuts, flaxseed and leafy vegetables- can potentiate AHN through at least two distinct mechanisms; i.e., affecting the membrane fluidity and improving the serotonin binding (a neurotransmitter that stimulates neurogenesis) or by regulating neurotrophins levels like BDNF (Wu et al., 2004; Beltz et al., 2007) since they are part of the structural neuronal membranes. Additionally, ω-3 FAs improve spatial learning abilities in mice, which is associated with enhanced neurogenesis (Petursdottir et al., 2008; He et al., 2009; Valente et al., 2009).

Folates—an important regulator of central nervous system (CNS) development present in dark green leafy vegetables, beans, peas and nuts- could plays a critical role in the maintenance of AHN by its DNA methylation and epigenetic functions (Kronenberg et al., 2008). Its deficiency diminishes the concentration of neurotransmitters in the hippocampus, affecting AHN (Kronenberg et al., 2008; Zhang et al., 2012). Another vitamin, cobalamin—found in meat, fish, poultry, eggs and dairy products- also plays an essential role in proper brain development and function, impairing its lack of AHN due to its action on DNA replication and methylation (Smith, 2016).

Polyphenols, the biggest group of phytochemicals found in plant-based foods, are known for their biological antioxidative, neuroprotective, and cognitive properties, considered as exogenous molecules able to modulate adult neurogenesis (Valente et al., 2009). Several studies suggest that polyphenols induce AHN by increasing synaptic plasticity and promoting long-term hippocampal potentiation (Xu et al., 2007; An et al., 2008; Valente et al., 2009; Wang et al., 2011) as well as enhance learning and memory (Van Praag et al., 2007; Duffy et al., 2008; Rendeiro et al., 2012). Flavonoids-enriched diets, a class of polyphenol present in plants and plant-based foods, demonstrated an increase in the number of newly generated cells expressing the immature neuron marker doublecortin and mature neuron markers (Valente et al., 2009; Lee et al., 2010; Dias et al., 2012) and in the expression of two essential factors closely related to hippocampal neurogenesis: BDNF and the phosphorylated cyclic AMP-response element DNA-binding protein (pCREB) (An et al., 2008; Zainuddin and Thuret, 2012; Tan et al., 2017).

All the aforementioned emphasizes the importance of informing the population about the possible implications of HCD on cognitive effects, promoting the consumption of diets rich in bioactive compounds. In addition, it would be convenient to encourage the study of new natural compounds that may increase the AHN in humans, thus being able to improve memory and learning in people with cognitive deficits.

There are few studies focused on describing sex differences in relation to the influence of diet on AHN; however, there seems to be a strong sexual dimorphism. Since the differences between male and female physiologies are greatly influenced by hormonal differences and these in turn influence adult neurogenesis, studies are needed that relate sex hormone levels to nutrition and their influence on AHN.

### 2.2. Role of the microbiota in the impact of diet on AHN

Recently, gut microbiota has been revealed as a key factor to regulate both AHN and signaling molecules relevant to neuroplasticity that are altered by diet. There is a close bidirectional communication between the brain and the gut through the brain-gut-microbiota axis, formed by the central nervous system, the hypothalamus-pituitary-adrenal axis, the endocrine-immune system, the autonomic nervous system, and the gut microbiota (Carabotti et al., 2015; Cryan, 2018). Due to this two-way communication, signaling from the gut can alter brain function and vice versa. In this sense, a contribution of the intestinal microbiota to neurogenesis has been demonstrated in recent years. Probiotic administration can ameliorate hippocampal neurogenesis decline (Corpuz et al., 2018). Diet supplementation with *Lactobacillus* strains in an animal mouse model of accelerated aging improves cognitive performance in some behavioral test. Moreover, this supplementation in old female mice showed augmented BDNF, CREB and p-CREB in the hippocampus (Corpuz et al., 2018). Conversely, antibiotic administration decreased hippocampal neurogenesis and induced changes in the molecular signaling related to this process. For example, animals exposed to antimicrobials show a general inflammatory state (Guida et al., 2018), reduced hippocampal BDNF, increased TrkB receptor expression, depressive-like behavior in male mice, and impaired novel object recognition (hippocampus-dependent memory) (Fröhlich et al., 2016; Guida et al., 2018). Nevertheless, prebiotics and probiotics administration reversed the negative influence of antibiotics in neurogenesis in female and male mice (Möhle et al., 2016; Guida et al., 2018).

Several factors such as antibiotics, stress and diet can alter the gut microbiota composition, changing AHN (Cryan, 2018; Ribeiro et al., 2020; Guzzetta et al., 2022). Regarding diet, one study reported that mice on a HFD evidenced significant changes in the gut microbiota composition, decreasing principally bacteria of phylum Bacterioidetes, which are correlated with lower neuroinflammation and higher BDNF levels and memory performance (Jørgensen et al., 2014). Other authors showed that a high-fat diet (choline-deficient diet) induced gut dysbiosis, increasing the production of short-chain fatty acids (propionate and butyrate) by gut microbiota. This change evocate neuroinflammation, oxidative stress, synaptic loss, cell death in different brain regions, and premature increased neurogenesis (Ribeiro et al., 2020). Moreover, an obesogenic diet (high fat and sucrose) showed altered hippocampus-dependent learning measured by the Morris water maze test and alteration in the gut microbial community in male mice (Sun et al., 2022). This obesogenic diet also increased the expression of proinflammatory cytokines that evocate neuroinflammation and altered AHN. However, the polysaccharides administration with prebiotic function could reverse the effect of this obesogenic diet, confirming that the modulation of gut microbiota is a therapeutic target for some neurodevelopmental disorders (Sun et al., 2022).

Altogether, these recent results demonstrate that microbial signaling can control neurogenesis and synaptic plasticity. Moreover, regulating the gut microbiota could be a therapeutic target for many disorders that evocate changes in AHN, since the modulation of the microbiome will regulate AHN. It is necessary to explore in depth the effects of the administration of prebiotics, probiotics and symbiotics on pathological conditions, in different clinical and preclinical studies. This will corroborate the positive effect of these food products, and the potential role of AHN as a mediator of these effects. Specially, these treatments may be relevant when there is the coexistence of other pathology or environmental factor that increases the vulnerability to a psychiatric disorder o neurodegenerative disease.

### 2.3. Caloric restriction and intermittent fasting

Different preclinical models reported increased AHN in intermittent fasting (IF) or caloric restriction (CR). Caloric restriction models generally refer to a 30–40% decrease in daily caloric intake whereas animal IF models are defined as *ad libitum* feeding/no access 8 h/16 h or 12 h/12 h paradigms without caloric restriction.

There is a strong consensus in that IF and CR promote AHN; for example by increasing the number of immature neuroblasts (Dias et al., 2021), increasing the survival of neuronal precursor cells and their differentiation into mature neurons (Lee et al., 2002; Levenson and Rich, 2007; Kaptan et al., 2015; Baik et al., 2020); boost the number of shuttle shaped cells in the subgranular zone, the cell density in CA3 and the number of neurons and glia (Bondolfi et al., 2004; Liu et al., 2017; Mana et al., 2017; Cao et al., 2022; Xu et al., 2022). Only one study evidenced a decreased number of new hippocampal neurons in response to IF (Setel et al., 2022).

Several authors suggest different mechanisms underlying IF- and CR-induced AHN. Both paradigms have been related to an overall increment of the expression of neurotrophins (Elesawy et al., 2021), such as neurotrophin-3 (Park et al., 2008, 2013; Pani, 2015), ciliary neurotrophic factor (Park et al., 2008) and BDNF (Park et al., 2008; Treccani et al., 2014; van Praag et al., 2014; Kaptan et al., 2015; Kim et al., 2015; Morgan et al., 2017; Li et al., 2020; Brocchi et al., 2022; Setel et al., 2022) especially in newly generated neurons of the dentate gyrus (Lee et al., 2002; Elesawy et al., 2021). Caloric restricted animals displayed increased expression of genes of neuronal protection and differentiation, such as NeuroD1 (Brandhorst et al., 2015; Li et al., 2020), Notch (Baik et al., 2020), Klotho (Dias et al., 2021), Egr1 (Hornsby et al., 2016).

Dietary restriction was also associated with epigenetic changes. Animals exposed to CR displayed attenuation of age-related CG/CH methylation and prevention of age-related hypermethylation (Hadad et al., 2018) while IF stimulated the inhibition of histone deacetylase (van Praag et al., 2014) and simultaneously the deacetylation of genes delaying processes of cellular aging (Landry and Huang, 2021). Additionally, CR was associated with dampened expression of versican; an age-related protein with an essential role in neuronal development, maturation and survival (Setel et al., 2022). Animals who followed a restricted diet presented an increase of microRNA MMV-MIR-713: CR increases gene ontology of predicted microRNA targets of generation of neurons, neuron differentiation, and development (Cicekdal et al., 2022). CR also raised the expression of the gene PARP, which is associated with DNA repair and chromatin remodeling (Setel et al., 2022).

Several studies highlighted that IF and CR dependent neurogenesis is linked with increased expression of hippocampal NPY (Singh et al., 2015; Cao et al., 2022; Xu et al., 2022) and depends on increased quantities of hippocampal acyl-ghrelin (Kim et al., 2015; Hornsby et al., 2016) and ghrelin receptor (Hornsby et al., 2016). Notably, IF and CR also induced a CREB-dependent increase in SIRT1 and SIRT3 (Pani, 2015; Liu et al., 2019; Baik et al., 2020; Landry and Huang, 2021). Little evidence also showed a role for GSK3β (Li et al., 2020).

CR activates gene Foxo3, which improves adult neurogenesis through the reduction of neuroinflammation (Pani, 2015; Kim et al., 2020). In this context, animals displayed a decrease of proinflammatory hormones and cytokines (van Praag et al., 2014), reduced activation of glia (Liu et al., 2017; Li et al., 2020) and increased IFN-γ (promoting neural differentiation and neurite outgrowth in neural adult stem cells) (Park and Lee, 2011).

Finally, IF and CR reduced hippocampal oxidative stress and total protein oxidation content by increasing catalase reactivity and superoxide dismutase activity (Singh et al., 2015; Ahn et al., 2019) along with the expression of heat shock protein 70 and glucose-related protein 78 (involved in oxidative stress protection) (Landry and Huang, 2021).

To sum up, current literature demonstrates that both CR and IF can promote AHN. Of course, this finding has pivotal importance because of its high translational value in clinical practice: it could be an intervention of simple implementation in clinical protocols and has little to none adverse effects. However, further studies aimed to discover the exact mechanisms by which CR and IF promote AHN and what mediators/pathways are directly involved are needed. Moreover, most of the studies were done in healthy adult male subjects: more research is needed to underline the role of CR and IF in AHN in models of neurodegenerative pathologies (i.e., Alzheimer’s disease), in females (to evaluate the effect of gender) and at different age points (childhood, adolescence, elderly) and in female individuals.

## 3. Effect of maternal diet on offspring AHN

The perinatal environment, from pre-conception to lactation, is especially vulnerable to adverse conditions such as malnutrition or overnutrition, being able to permanently alter brain structure and function in the offspring. Thus, nutritional programming is partly responsible for cognition-related diseases in the offspring, even though clinical signs may only first arise in adulthood (Murray et al., 1991; Bolton and Bilbo, 2014; Rivera et al., 2020).

Adult hippocampal neurogenesis is sensitive to diet-related damage from direct consumption as we mentioned above (section 2.1) or from maternal exposure (Niculescu and Lupu, 2009; Mendes-da-Silva et al., 2014; Poulose et al., 2017). Maternal overfeeding during pregnancy and/or lactation greatly impacts adult neurogenesis in offspring (Niculescu and Lupu, 2009; Tozuka et al., 2009; Lépinay et al., 2015; Xavier et al., 2021). In this sense, consumption of a HFD during pregnancy leads to long-term effects in the central nervous system of the offspring, affecting neurogenesis-related pathways (Mash1 and BDNF) which correlated with the development of anhedonic-like behavior in the adulthood (Curi et al., 2021). Furthermore, perinatal exposure to HFD sensitizes the offspring to the adverse effects of postnatal high-fat intake on hippocampal function. This sensitization decreases AHN and reduces the expression of genes involved in hippocampal plasticity (Lépinay et al., 2015). Interestingly, it has been shown that the metabolic stress caused by the maternal consumption of HFD has a persistent influence, exerting multigenerational effects (up to the second and third generations) in the adult neurogenesis of their descendants; through an epigenetic disorder of pro-neurogenic genes in neural stem/progenitor cells (NSPC) (Natale et al., 2022).

Maternal consumption of a high-fructose diet during pregnancy and lactation decreased BDNF and suppressed hippocampal expression of Ki67 and DCX. These markers are related to NSPC division and neuronal differentiation; impairing hippocampal learning and memory in adult female offspring (Wu et al., 2016; Liu et al., 2020). These findings add to evidence suggesting that increased nuclear histone deacetylase 4 (HDAC4) activity induced by high-fructose maternal diets suppresses hippocampal neurogenesis in adult offspring.

Interestingly, few studies describe the impact of perinatal caloric restriction on offspring neurogenesis. Cell proliferation in the DG was significantly reduced in the offspring of mothers with 50% caloric restriction during pregnancy and/or lactation (Matos et al., 2011). Another study establishes an indirect relationship, demonstrating that the total and specific expression of the Igf2 allele (which regulates development, memory, and AHN) of the hippocampus is affected by maternal and grandmaternal moderate caloric restriction in a sex-specific manner (Harper et al., 2014).

In relation to this, prenatal protein restriction (8% protein diet during gestation and lactation) also reduces neuronal proliferation and BDNF expression in adult offspring, and is also associated with problems with memory encoding and consolidation (Pérez-Garciá et al., 2016).

It is well known that there are beneficial bioactive compounds during pregnancy and lactation for both the mother and the developing fetus; thus, many nutrients such as iron, zinc, selenium, iodine, folic acid, vitamin A, vitamin B6, vitamin B12 and choline are essential for neurodevelopment. Interestingly, some studies indicate that some of these compounds also improve the neurogenesis of offspring in adulthood; for example, prenatal iron deficiency results in long-term memory deficits (Lucassen et al., 2013); postnatal zinc supplementation improves cognitive impairment induced by zinc deficiency in early life, associated with abnormal expression of genes involved in DNA methylation and neurogenesis (Jiang et al., 2022).

Thus, maternal choline supplementation has been shown to improve cognitive function by increasing AHN in the offspring in both normal animals and animal models of neuropathologies such as Down syndrome and Alzheimer’s disease (Glenn et al., 2007; Cheng et al., 2008; Wong-Goodrich et al., 2008; Moon et al., 2010; Velazquez et al., 2013). Maternal supplementation with nicotinamide riboside (RN) during lactation also increase AHN in the offspring in rodents, which may be responsible for the neurobehavioral improvement, through enhanced nicotinamide adenine dinucleotide (NAD+) metabolism (Ear et al., 2019; Yang and Wan, 2019).

Maternal folic acid supplementation also promotes hippocampal neurogenesis and improves learning and memory in offspring, implicating mechanisms associated with DNA methylation in glucocorticoid receptor promoters and activation of BDNF/AKT/ERK1/2 signaling (Yang et al., 2019).

Preclinical studies have improved the understanding of the underlying mechanisms linking maternal intake and offspring neurodevelopment. In this sense, and in relation to what was previously described in section 2.2, the microbiota-gut-brain axis acts as a fundamental regulator of neurodevelopment (Dinan and Cryan, 2017; Ratsika et al., 2021). Maternal diet affects the composition of the maternal and neonatal gut microbiome in rodents, associated with abnormalities in brain function and behavior of the offspring (Paul et al., 2016; Vuong et al., 2020). Intake of a probiotic supplement during pregnancy and lactation has a long-lasting influence on behavior and neuroplasticity, mitigating anxiety-like behavior associated with maternal obesity (prolonged high-fat diet) in adult offspring. These improvements are associated with an increase in the expression of BDNF and other genes related to plasticity in adulthood of the offspring (Radford-Smith et al., 2022).

The findings summarized here confirm the importance of nutrition in neuronal plasticity from the perinatal stage. The mental health of an individual depends not only on himself but also in part on his progeny. However, this knowledge is not yet entrenched in society as there is still no great evidence at the clinical level. Thus, more studies in humans where maternal, and probably paternal, nutritional states are correlated with long-term cognitive alterations in offspring are necessaries. Those data has been difficult to collect up to now due to the absence of computerized records of the perinatal stage. Another limitation at the clinical level compared to animal models is the difficulty of studying AHN directly. For this reason, the gut-brain connection through the study of the microbiota opens an interesting topic of study, not only in adult individuals but also from their gestation. The analysis of the microbiota in relation to nutrition and its correlation with genes related to neuronal plasticity and plasmatic neurogenic markers will provide great information on the mechanisms involved in neuroplasticity and will open both preventive and therapeutic action pathways against cognitive disorders and neurodegenerative diseases.

## 4. Conclusion

The current literature provides solid evidence about the fundamental role of dietary factors in hippocampal plasticity. Malnutrition from the fetal stage and/or throughout life contributes to the acceleration of age-related deterioration, positioning diet as an important factor in the risk, progression, and severity of the mental diseases. It can be concluded that an adequate dietary intake from the perinatal stage to adulthood, considering quantity, frequency and content of food or bioactive compounds, should be considered and promoted as a public health initiative for the prevention and improvement of neuropsychiatric disorders.

Modulation of AHN by diet could emerge as a possible mechanism by which nutrition impacts on mental health. Despite the knowledge of the large implication of factors such as BDNF, and the promising role of factors such as gut microbiota, further investigation of the mechanisms by which prenatal and early postnatal life nutritional factors influence AHN -and consequently cognitive function- would contribute to understanding this relationship and would have important implications for dietary modification of brain’s response to injury and disease. Nevertheless, research on AHN in humans is still at an early stage and more translational research is needed to confirm a functional role of AHN in the human brain as well as its modulation by diet.

Most of the studies reviewed here do not contemplate the possible differentiating effect of diet on the AHN of male and female animals. In general, studies based on nutritional programming on offspring AHN include both male and female offspring, but even so, some of them do not focus on possible sexual dimorphism. However, studies on the nutrition-AHN relationship that include male and female animals show sex-specific dietary effects, with females generally being more susceptible.

## Author contributions

EC-O, AC-Z, and PR designed the study. All authors performed the research, wrote the manuscript, have made a substantial, direct, and intellectual contribution to the work, and approved it for publication.
